# Oligomerization of Cu,Zn-Superoxide Dismutase (SOD1) by Docosahexaenoic Acid and Its Hydroperoxides *In Vitro*: Aggregation Dependence on Fatty Acid Unsaturation and Thiols

**DOI:** 10.1371/journal.pone.0125146

**Published:** 2015-04-30

**Authors:** Patricia Postilione Appolinário, Danilo Bilches Medinas, Adriano B. Chaves-Filho, Thiago C. Genaro-Mattos, José Renato Rosa Cussiol, Luis Eduardo Soares Netto, Ohara Augusto, Sayuri Miyamoto

**Affiliations:** 1 Departamento de Bioquímica, Instituto de Química, Universidade de São Paulo, São Paulo, Brazil; 2 Departamento de Biologia, Instituto de Biociências, Universidade de São Paulo, São Paulo, Brazil; University of Florida, UNITED STATES

## Abstract

Docosahexaenoic acid (C22:6, *n*-3, DHA) is a polyunsaturated fatty acid highly enriched in the brain. This fatty acid can be easily oxidized yielding hydroperoxides as primary products. Cu, Zn-Superoxide dismutase (SOD1) aggregation is a common hallmark of Amyotrophic Lateral Sclerosis (ALS) and the molecular mechanisms behind their formation are not completely understood. Here we investigated the effect of DHA and its hydroperoxides (DHAOOH) on human SOD1 oligomerization *in vitro*. DHA induced the formation of high-molecular-weight (HMW) SOD1 species (>700 kDa). Aggregation was dependent on free thiols and occurred primarily with the protein in its apo-form. SOD1 incubation with DHA was accompanied by changes in protein structure leading to exposure of protein hydrophobic patches and formation of non-amyloid aggregates. Site-directed mutagenesis studies demonstrated that Cys 6 and Cys 111 in wild-type and Cys 6 in ALS-linked G93A mutant are required for aggregation. In contrast, DHAOOH did not induce HMW species formation but promoted abnormal covalent dimerization of apo-SOD1 that was resistant to SDS and thiol reductants. Overall, our data demonstrate that DHA and DHAOOH induce distinct types of apo-SOD1 oligomerization leading to the formation of HMW and low-molecular-weight species, respectively.

## Introduction

Amyotrophic Lateral Sclerosis (ALS) is a progressive and fatal disease caused by early and selective degeneration of motor neurons [1, 2]. ALS is predominantly sporadic, with approximately 5–10% of familial cases (fALS) [3]. Around 20% of fALS cases are due to mutations in the gene encoding the cytosolic antioxidant enzyme superoxide dismutase 1 (SOD1) [3].

Many mutations (>170) in Cu,Zn-superoxide dismutase (SOD1) have been described in fALS (http://alsod.iop.kcl.ac.uk/). The ALS-linked mutations have been associated to a gain of toxic functions by the enzyme, including the formation of cytotoxic oligomers and aggregates [4–6]. Molecular mechanisms involved in the SOD1 aggregates formation are still unclear *in vivo*, however studies accumulated over the years have uncovered some key factors triggering its aggregation, such as metal mishandling [7, 8], abnormalities in thiol-disulfide status [9], and oxidative modifications [10–12]. Relevantly, in this context, some lipids have been shown to enhance oligomerization of proteins involved in neurodegeneration, including α-synuclein, β-amyloid, prion protein, and SOD1 [13–22].

Docosahexaenoic acid (C22:6 *n*-3, DHA) is a omega-3 fatty acid present in high concentrations in the brain gray matter where it accounts for approximately 50% of the total polyunsaturated fatty acid content in cell membranes [23, 24]. Although most of DHA is found esterified to phospholipids in cell membranes, its release can be increased under inflammatory conditions by phospholipase A2 [25, 26]. In support of this hypothesis, studies have shown that ALS is intimately linked with neuroinflammation [26–29], a condition in which free DHA could be increased.

DHA is known to be oxidized enzymatically and/or non-enzymatically to hydroperoxy- and hydroxy-derivatives [30]. Great attention has been given to the enzymatically oxygenated DHA derivatives especially due to their neuroprotective and anti-inflammatory properties [25, 31]. However, less in known about the properties of the non-enzymatic oxidation products (e.g. hydroperoxides) formed upon oxidation of DHA by reactive oxygen species.

Considering the abundance of DHA in the brain and its high propensity to oxidation, here we aimed to investigate the effects of DHA and its hydroperoxides (DHAOOH) on human SOD1 aggregation *in vitro*. Using metal-deficient SOD1 WT and G93A we have confirmed the ability of polyunsaturated fatty acids to induce SOD1 aggregation [22]. Moreover, we have advanced the understanding on the mechanism involved in this process, by showing the dependence of the aggregation on specific thiol groups in the protein and also on the *cis* conformation of the unsaturated bond in the fatty acid. In contrast, DHAOOH showed minor effects on large aggregate species formation. Alternatively, the interaction of apo-SOD1 with DHAOOH induced a different type of modification that lead to the formation of small low molecular weight oligomers (e.g. dimers) resistant to thiol reductants.

## Materials and Methods

### Materials

Amicon ultra centrifugal filters were obtained from Merck-Millipore (Merck Millipore, Germany). Bis-ANS (4,4'-dianilino-1,1'-binaphthyl-5,5'-disulfonic acid, dipotassium Salt) and iodoacetamide-fluorescein (IAF) were from Molecular Probe (Life Technologies do Brasil Ltda). Bromophenol blue, tetramethylethylenediamine (TEMED), molecular weight marker Kaleidoscope, glycerol, glycine, acrylamide were obtained from Bio-Rad Laboratories (CA, USA). Acids and solvents (HPLC grade) were from J.T. Baker (Avantor Performance Materials, Mexico). All other reagents were from Sigma (St. Louis, MO). All aqueous solutions were prepared with ultrapure water purified by a Direct-Q3 system (Merck Millipore, Germany) and treated with Chelex 100 before use.

### DHAOOH preparation

DHAOOH was prepared as previously reported by photosensitized oxidation in the presence of methylene blue [30, 32]. Briefly, synthesis of DHAOOH was performed by photosensitization reaction by adding 4 μL of a solution of methylene blue (0.1 M in methanol) to 25 mg docosahexaenoic acid dissolved in 4 mL of chloroform (~ 20 mM). The mixture was kept under constant stirring, saturated with oxygen and irradiated with two tungsten lamps (500 W) for approximately 2 h. DHAOOH was purified as a mixture containing 12 isomers by semi-preparative C18 column (ThermoQuest 250 x 10mm, 10μm size particle) and mobile phase 55% acetonitrile containing 0,005% formic acid at a flow rate of 4,7 mL/min. Hydroperoxides were checked by mass spectrometry and quantified by absorbance at 234 nm (ε = 25200 M^-1^cm^-1^) and iodometry [30].

### Human recombinant SOD1 expression and purification

The enzyme was expressed in *Escherichia coli* and purified as previously described [33]. Purified protein was repeatedly washed and concentrated by ultrafiltration filter cutoff 30 kDa (Amicon Ultra Centrifugal Filter) in 5 mM phosphate buffer, pH 7,4, treated with Chelex -100.

### Preparation of apo-SOD1 WT and apo-SOD1 G93A

Apo-forms were prepared from SOD1 by repeated dialysis against: (1) 50 mM acetate buffer, pH 3.8 containing 10 mM EDTA, (2) 50 mM acetate buffer, pH 3.8 containing 100 mM NaCl to remove EDTA and (3) finally, against Milli-Q grade water, treated with Chelex-100 resin to remove traces of transition metals [34].

### SOD1 oligomerization experiments

Soluble apo-SOD1 WT and/or G93A was dissolved in 50 mM phosphate buffer pH 7.4 containing 150 mM NaCl and 100 μM of the iron chelator diethylenetriaminepentaacetic acid (DTPA). Oligomerization studies were performed by incubating 10 μM of apo-SOD1 WT or G93A with 250 μM of fatty acids (DHA, stearic acid, oleic acid, elaidic acid, linoleic acid or arachidonic acid), DHAOOH or H_2_O_2_ at 37°C for 24 h. Time dependent analysis was done by incubating the samples for 2, 6 and 24 h. Dose dependent experiments were done with 50, 100 and 250 μM DHA or DHAOOH at 37°C for 24 h.

### SDS-PAGE

SDS-PAGE analysis was performed under reducing and non-reducing using a 12% polyacrylamide gel. After incubation, aliquot (20 μL) of the samples was pre-incubated with 50 mM iodoacetic acid sodium salt (NaIAc) for 15 min, and then treated with SDS-PAGE sample buffer (62 mM Tris-HCl, pH 6.8 containing 10% glycerol, 2% SDS, 0.01% bromophenol blue) in the absence or presence of β-mercaptoethanol (β-ME), boiled for 5 min and subjected to electrophoresis in denaturing polyacrylamide gels (5% stacking gel, 12% resolving gel). Gels were stained with silver nitrate.

### Size exclusion chromatography

Size exclusion chromatography was performed using BioSep-SEC-S4000 column (300 x 7.8 mm, Phenomenex, USA). The size of SOD1 aggregates was determined using as standards the protein thyroglobulin (660 kDa), ferritin (440 kDa), aldolase (158 kDa), conalbumin (75 kDa) and ovalbumin (43 kDa). Each sample was eluted with a 50 mM phosphate buffer, pH 7.4 containing 150 mM NaCl and 100 mM DTPA. Proteins were analyzed at 210 nm.

### Congo red (CR) assay

This method is based on red shift of the CR absorbance peak that is characteristic of CR binding to amyloid fibers by scanning from 400 nm to 650 nm in UV/Vis spectrophotometer [35]. For the analysis 30 μL of the sample incubated as described in the oligomerization experiments was mixed with 20 μL of 30 μM CR solution (final concentration 6 μM) and 50 μL of 5 mM phosphate buffer pH 7.4. Absorption spectra were acquired with a Cary 50 Bio UV/visible spectrophotometer (Varian).

### Bis-ANS fluorescence

Exposure of protein hydrophobic patches was verified after 24 h incubation by analyzing the fluorescence of the dye bis-ANS. An aliquot of 60 μL of each sample was incubated with 12 μL of bis-ANS (60 μM) for 15 minutes in water at 37°C. The fluorescence spectrum was obtained by a plate reader (TECAN, Life Technologies). Emission was recorded in the range of 400–650 nm after excitation at 390 nm. Baseline measurements of the bis-ANS in the presence of DHA, DHAOOH or H_2_O_2_ were taken as a control. After that, fluorescence measurements of bis-ANS in the presence of apo-SOD1 WT or G93A incubated with DHA, DHAOOH or H_2_O_2_ was recorded.

### Transmission electron microscopy (TEM)

Aggregate morphology was analyzed by TEM as described previously [36]. Briefly, two microliters of the samples containing aggregates were adsorbed for 5 min onto on 200-mesh carbon-coated copper grids (Electron Microscopy Sciences EMS, Pennsylvania). After drawing off excess solution, the grids were air-dried and then stained with 2% (wt/vol) uranyl acetate. The specimens were viewed with a FEG-SEM JEOL JSM-7410 microscope at an accelerating voltage of 30 kV as described previously.

### Site-directed mutagenesis

Mutations were performed using the QuikChange kit standard site-directed mutagenesis protocol (Stratagene, Agilent Technologies, Inc., Santa Clara, California, USA). Two oligonucleotides (each complement its opposite strand) containing the mutation of interest were synthesized. The following primers were used: C6S: forward 5’- ACTAAAGCTGTGTTCGTGCTGAAGGGCGAC-3’; reverse 5’-GTCGCCCTTCAGCACGAACACAGCTTTAGT-3’; C111S: forward 5’- TCTCAGGAGACCATTCCATCATTGGCCGCA-3’; reverse 5’-TGCGGCCAATGATGGAATGGTCTCCTGAGA-3’. The mutagenesis products were transformed into XL1-Blue by electroporation and plated in solid selective LB/Amp. The resulting colonies were selected for confirmation of the insert by PCR and amplification was performed using T7 *primers* flanking the region of interest in the vector (pET-3d). After this, colonies in which the insert was confirmed were grown in liquid LB/Amp. The coding region of the isolated vector was sequenced to verify the mutations generation and the vectors with their confirmed mutations were transformed into strains of *E*. *coli* expression BL21(DE3)pLysS.

## Results

### SOD1 oligomerization in the presence of DHA and its hydroperoxides

Incubations of metal-deficient SOD1 (apo-SOD1 wild-type and mutants) with unsaturated fatty acids (e.g. arachidonic acid) has been previously shown to induce the formation of high molecular weight (HMW) oligomers [22]. To characterize how fatty acids in the brain could affect SOD1 aggregation, we focused our investigation on the ability of DHA and its oxidized counterpart, the monohydroperoxides (DHAOOH, a primary product of DHA oxidation) to induce SOD1 oligomerization. Experiments were conducted with the protein in its apo-form since this form was the most susceptible to undergo aggregation. Moreover, apo-SOD1 is found enriched in insoluble SOD1-rich fractions in ALS transgenic mice spinal cords [8, 37] and likely to constitute an important form of SOD1 in familial and sporadic ALS [7, 38–41].

**Fig 1:**
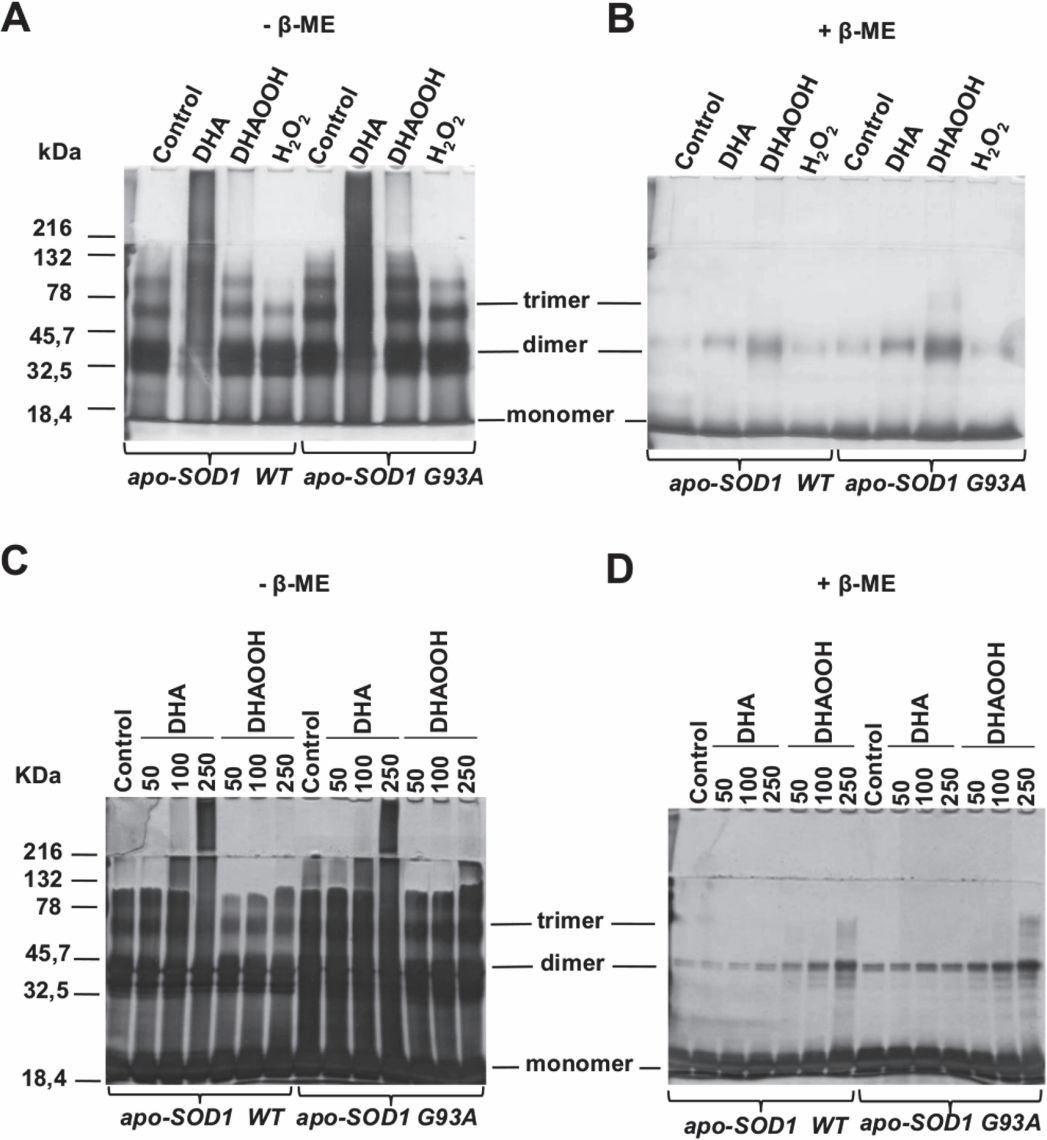
Apo-SOD1 oligomerization in the presence of DHA and DHA hydroperoxides. Apo-enzymes of SOD1 WT or G93A mutant (10 μM) were incubated in the presence of 250 μM DHA, DHAOOH or H_2_O_2_ at 37°C for 24 h and then analyzed by SDS-PAGE under non-reducing (A) and reducing conditions (B). Similarly, concentration dependent analysis of SOD1 oligomerization was assayed by incubating the proteins with 50, 100 or 250 μM of DHA or DHAOOH and analyzed by SDS-PAGE under non-reducing (C) and reducing (D) conditions. Gels in A and B are representative of more than 3 experiments and gels in C and D are representative of two experiments.

DHA or DHAOOH was incubated with both WT and G93A apo-SOD1 at 37°C for 24 h and oligomerization was assessed by SDS-PAGE under non-reducing and reducing conditons (Fig 1). Interestingly, they have induced distinct effects on protein oligomerization (Fig 1A and 1B).

DHA induced SOD1 aggregation as shown by the appearance of HMW species of the protein under non-reducing conditions (Fig 1A). Aggregation was slightly more pronounced in the G93A mutant compared to WT which is in accordance with the more unstable nature of the mutant. Importantly, SOD1 HMW aggregates were completely reversed in the presence of β-mercaptoethanol (β-ME) (Fig 1B), indicating that SOD1 aggregates were disulfide bonded.

On the other hand, DHAOOH had minor effects on SOD1 HMW aggregate formation as can be observed in non-reducing gels (Fig 1A). Alternatively, DHAOOH induced the formation of abnormal dimeric species, which can be observed by the increase of a band at around 32 kDa in the reducing gels. For comparison, we tested the effect of H_2_O_2_, which had no effects on both aggregation and dimerization. Dimeric species were also not formed by *tert*-butylhydroperoxide (data not shown). The resistance of dimeric SOD1 species to thiol reductants was confirmed with guanidine (2 M) and dithiothreitol (166 mM) (S1 Fig).

Dose-dependent effects of DHA and DHAOOH on SOD1 oligomerization were studied. Incubations conducted with 50 μM, 100 μM and 250 μM of DHA or DHAOOH showed a dose-dependent increase on both aggregation (Fig 1C) and dimerization (Fig 1D), under non-reducing and reducing conditions, respectively. Again, the effects of DHA and DHAOOH were more pronounced with the G93A mutant form of the enzyme.

**Fig 2:**
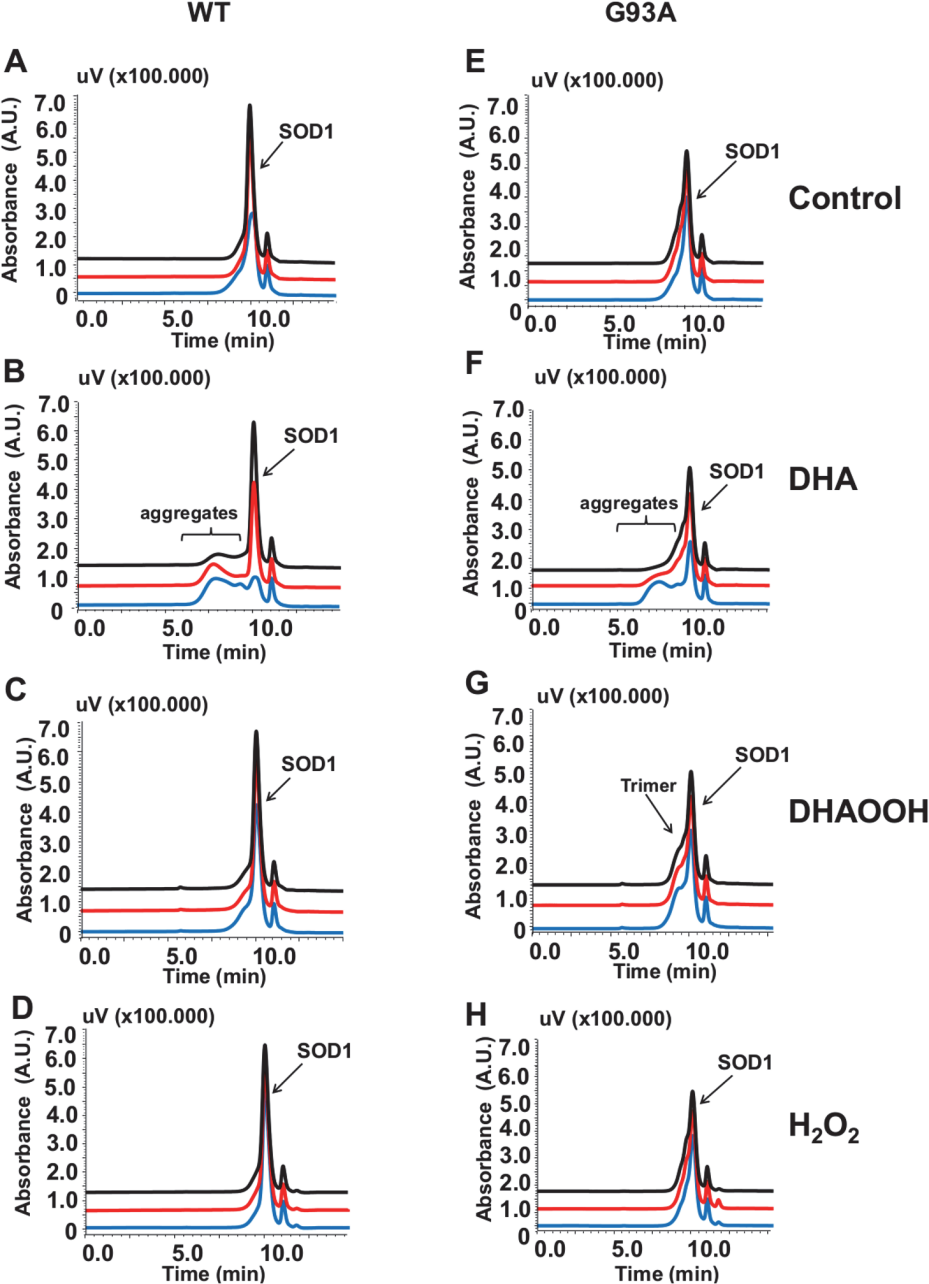
Analysis of apo-SOD1 aggregates by size-exclusion chromatography. Apo-SOD1 WT incubated in the absence (**A**) and presence of 250 μM of DHA (**B**), DHAOOH **(C)** or H_2_O_2_ **(D)**. Apo-SOD1 G93A incubated in the absence **(E)** and presence of 250 μM of DHA **(F)**, DHAOOH **(G)**, or H_2_O_2_ **(H)**. All experiments were conducted in the presence of 10 μM of the protein at 37°C for 24 h. The colored lines represent the incubation times: 2 h (black), 6 h (red) and 24 h (blue). Chromatograms are representative at least 3 independent experiments.

SOD1 oligomerization was also checked by size-exclusion chromatography (Fig 2). In line with the results observed in SDS-PAGE analysis, DHA promoted the formation of large aggregate species (>700 kDa) (Fig 2). Peaks corresponding to the HMW aggregate species could be observed as early as 2 h after incubation with both proteins (Fig 2B and 2F). For the incubations with DHAOOH it was possible to notice a broadening of the native SOD1 peak (Fig 2C and 2G) possibly related to the formation of abnormal SOD1 dimers and trimer species.

### Exposure of protein hydrophobic patches and formation of amorphous aggregate species

To verify whether DHA and DHAOOH induce alterations in protein hydrophobicity we conducted experiments with bis-ANS, a probe that has increased fluorescence in hydrophobic environments [42]. In addition, we examined the formation of amyloid-like fibrillar aggregates by the CR assay and by TEM analysis.

**Fig 3:**
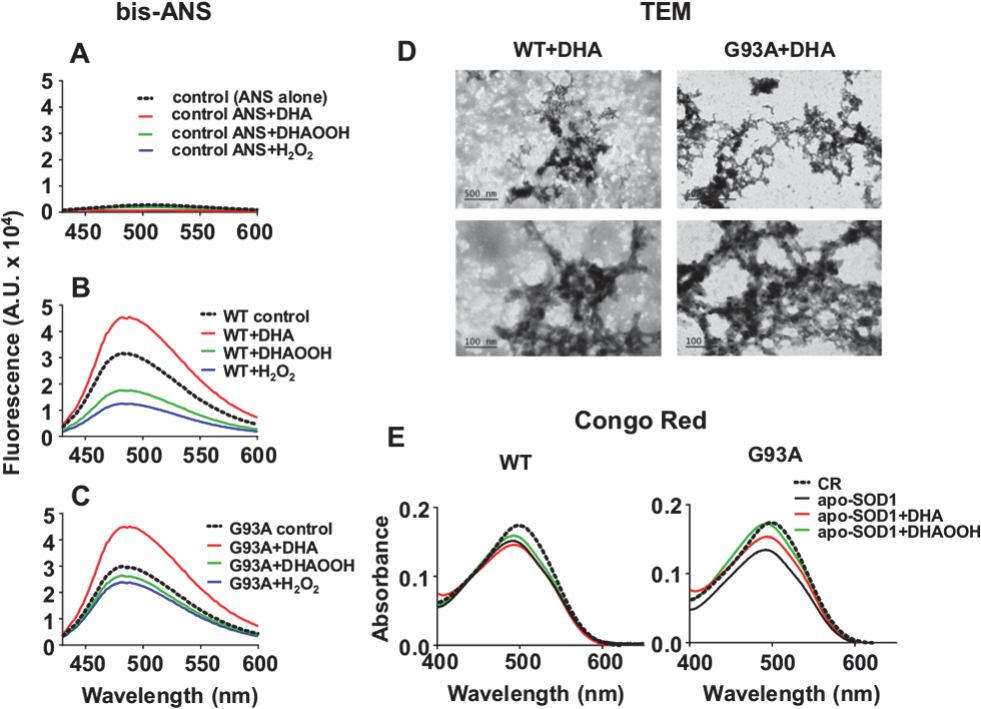
Nature of the apo-SOD1 aggregates formed in the presence of DHA or DHAOOH. Representative bis-ANS fluorescence spectra obtained for the control incubations containing DHA, DHAOOH or H_2_O_2_ without the protein (A); and for the incubations containing the (B) apo-SOD1 WT or (C) apo-SOD1 G93A incubated in the presence of DHA, DHAOOH or H_2_O_2_. Transmission electronic microscopy (TEM) of the apo-SOD1 WT or G93A mutant incubated with DHA (D). Two sets of images, one with the scale bar of 500 nm (upper panel) and the other of 100 nm (lower panel) are shown. Images are representative of two experiments. Representative visible spectra of congo red (CR) in the absence and presence of apo-SOD1 WT or G93A mutant pre-incubated with DHA or DHAOOH. Samples were added to the CR solution to give a final concentration of 6 μM of CR. All incubations were conducted in the presence of 10 μM protein and 250 μM of the lipid or H_2_O_2_ at 37°C for 24 h.

The fluorescence of bis-ANS probe showed the characteristic blue shifted fluorescence spectrum whose intensity was augmented for the incubations of WT or G93A apo-SOD1 with DHA, indicating an increase in protein hydrophobicity (Fig 3B and 3C). On the contrary, in the presence of DHAOOH or H_2_O_2_, bis-ANS fluorescence was lower than control containing only the protein (Fig 3B and 3C). This effect could be interpreted by increased polarity of the protein surface possibly related to the oxidation of solvent-exposed aminoacid residues by the hydroperoxides.

Amyloid-bound CR is known to display a red shift in the UV absorbance from about 490 to 540 nm [43]. Spectra recorded for the CR alone and CR incubated in the presence of apo-SOD1 WT or G93A pre-incubated with DHA or DHAOOH for 24 h showed a slight blue-shift in relation to CR alone. However, none of the samples promoted the red shift of the CR absorbance peak that is characteristic of CR binding to amyloid (Fig 3E), thus suggesting that the aggregates formed in the presence of the fatty acids are nonamyloid. Accordingly, TEM analysis of both apo-SOD1 WT and apo-SOD1 G93A incubated with DHA for 24 h presented amorphous aggregates and no clear amyloid fibers (Fig 3D). Images show aggregates composed by granular shape components with approximately 10 nm diameter.

### Protein oligomerization dependency on fatty acid unsaturation and conformation

**Fig 4:**
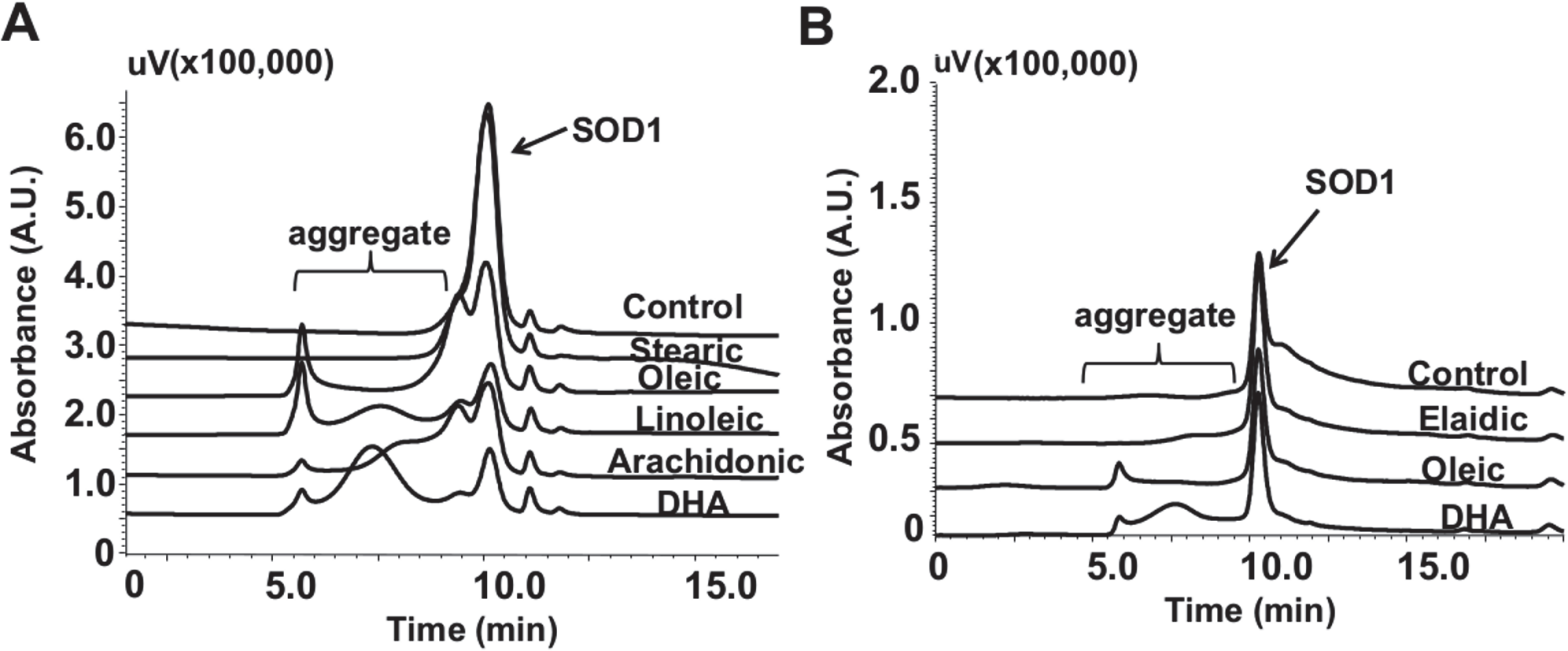
Aggregation of apo-SOD1 WT and G93A in the presence of different fatty acids analyzed by size-exclusion chromatography. Typical chromatograms obtained for the incubations of apo-SOD1 WT (A) or G93A mutant (B) with different fatty acids. All incubations were performed with 10 μM of the protein and 250 μM of each fatty acid at 37°C for 24 h. Arrows indicate the SOD1 main peak and brackets indicate the SOD1 oligomers. Chromatograms are representative of at least 3 independent experiments.

To explore the relationship between aggregation and number of unsaturated bonds in the fatty acid, we incubated apo-SOD1 WT or G93A with stearic acid (18:0), oleic acid (18:1), linoleic acid (18:2), arachidonic acid (20:4) or DHA (22:6) (Fig 4). Among these fatty acids, only the unsaturated ones were capable to induce protein aggregation. We further expanded these observations by also testing whether fatty acid conformation would affect SOD1 oligomerization. For this purpose, we compared the effect of oleic acid and the corresponding fatty acid in the *trans* configuration, the elaidic acid (Fig 4B). Notably, aggregation occurred only with the fatty acid with the unsaturated bond in the *cis* configuration.

### Participation of cysteines 6 and 111 on apo-SOD1 oligomerization

**Fig 5:**
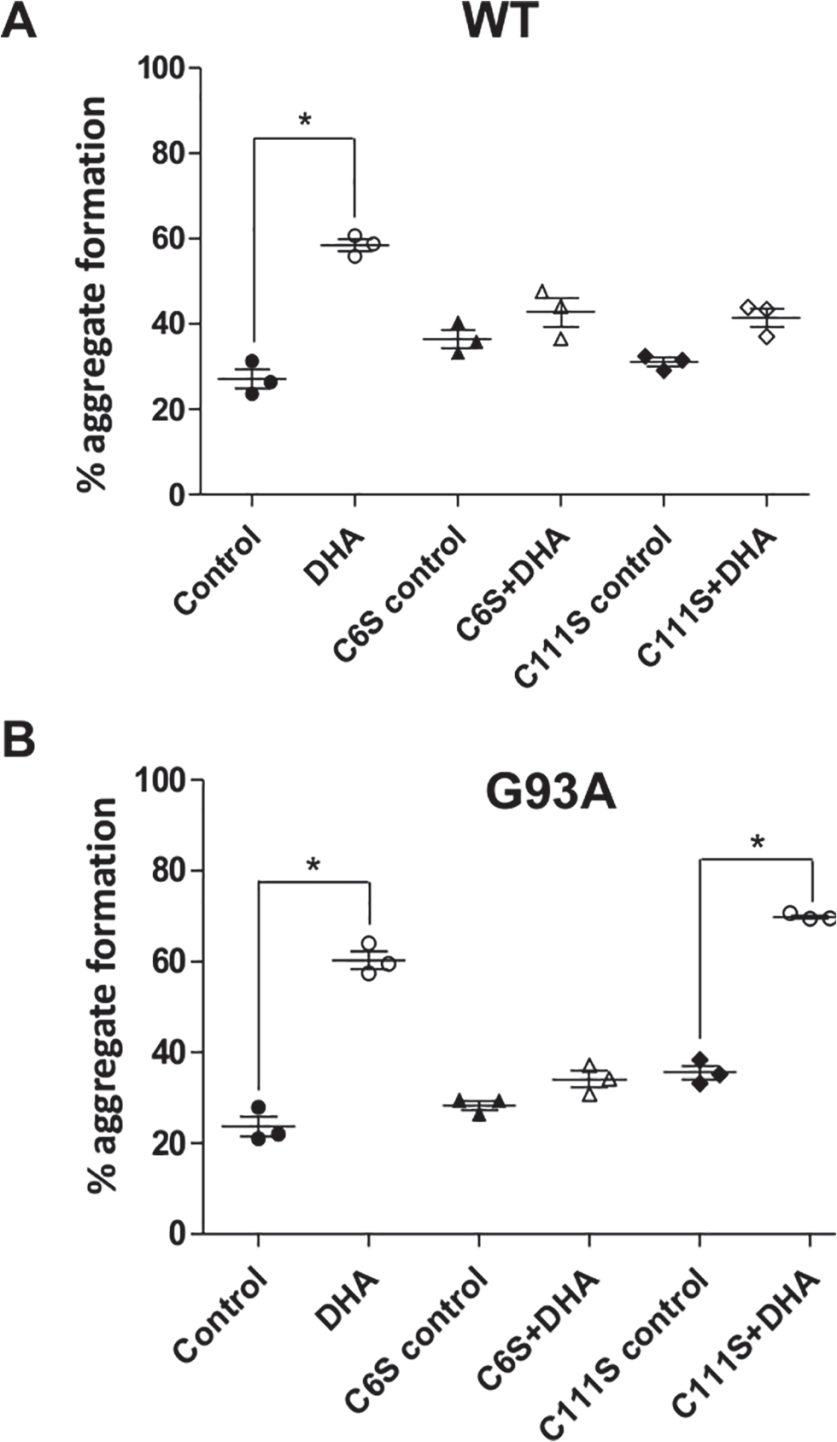
Role of Cys 6 and Cys 111 on DHA induced apo-SOD1 aggregation. C6S and C111S mutants of apo-SOD1 (10 μM) WT (**A**) and G93A (**B**) were incubated in the absence and presence of DHA (250 μM). Percentages of aggregates formed in the incubation were determined by size-exclusion chromatography analysis. The results were presented by means ± standard deviations of three experiments. Significant differences are indicated with * when *p<0*.*01*. Dots represent individual values.

Cys-6 and Cys-111 in SOD1 have distinct localization and solvent accessibilities [44–46]. Site-directed mutagenesis studies were performed to check which of these free Cys residues participate in the aggregation process induced by DHA. The apo-SOD1 mutants, C6S and C111S of both WT and G93A, were prepared and then incubated in the absence and presence of DHA. The percentages of protein that underwent oligomerization were analyzed by size-exclusion chromatography (Fig 5A and 5B).

For the apo-SOD1 WT, both C6S and C111S mutations inhibited aggregation (Fig 5A), suggesting that Cys 111 and Cys 6 might act synergistically in the oligomerization process. Conversely, for the apo-SOD1 G93A mutant (Fig 5B), only C6S was capable to inhibit DHA induced aggregation, indicating that Cys 6 is particularly important in the aggregation mechanism induced in the presence of the fatty acid.

## Discussion

Previous studies [19, 22] showed that SOD1 oligomerization could be induced by lipid molecules. Given the high propensity of polyunsaturated fatty acids to oxidation, we aimed to evaluate the effect of DHA and its derived hydroperoxides on SOD1 oligomerization. We have initially postulated that hydroperoxides would enhance oligomerization by promoting protein oxidation, a process that has been described to contribute in the formation of unfolded/misfolded SOD1 forms [47]. However, oligomerization promoted by the DHAOOH was modest when compared to DHA itself, as corroborated by SDS-PAGE (Fig 1) and size-exclusion chromatography analysis (Fig 2).

A closer examination of apo-SOD1 oligomerization by non-reducing and reducing SDS-PAGE (i.e. without and with β-ME, Fig 1A and 1B) revealed that DHA and its oxidized counterpart produce distinct types of cross-links in apo-SOD1 that are, respectively, sensitive and resistant to reducing agents such as β-ME. As a result, HMW aggregates and abnormal covalent dimeric species were produced with DHA and DHAOOH, respectively. Accordingly, protein unfolding analyses using bis-ANS indicate that DHA induces increase in protein surface hydrophobicity possibly due to the exposition of hydrophobic residues. In contrast, DHAOOH was unable to promote increases in protein hydrophobicity and subsequent large aggregate formation. Reasons for that could be the oxidation of protein residues involved in the oligomerization process. For instance, hydroperoxides can oxidize thiols to sulfinic (-SO_2_H) and sulfonic acids (-SO_3_H) thereby avoiding the formation of intermolecular disulfide bonds [6, 48]. Alternatively, the presence of a peroxide group in the acyl chain could somehow affect proper lipid-protein interactions necessary for the protein aggregation.

Protein aggregates commonly found in many neurodegenerative diseases are classified as amyloid or non-amyloid based on a number of parameters including spectroscopic and dye based assays [35]. Here, we investigated the morphological nature of the aggregates formed upon DHA incubation by CR assay and TEM analysis (Fig 3). Incubation of CR with WT and G93A mutant aggregates did not cause the typical CR spectrum red shift observed for amyloid fibrils, suggesting a non-amyloid nature of the aggregate. This was confirmed by TEM analysis, which showed aggregates of amorphous morphology composed by granular shape substructures (Fig 3D). Interestingly, these data are consistent with the morphology found in previous studies on SOD1 aggregation promoted by lipids [19, 22]. More importantly, they resemble those aggregates found in ALS patients [49, 50] and in early aggregates found in animal and *in vitro* models [43, 51].

To get some insights on the mechanism by which DHA induces apo-SOD1 oligomerization, we evaluate the effect of different fatty acids containing varying degrees of unsaturation and double configurations (*cis* and *trans*). Our data confirmed that apo-SOD1 oligomerization occurs with fatty acids containing at least one unsaturated bond (e.g. oleic acid) [22]. Moreover, the nature (*cis* or *trans*) of the fatty acid double bond also affected protein oligomerization. HMW aggregates were not observed with the *trans* monounsaturated fatty acid. This could be due to differences in the biophysical properties of the fatty acids in solution, in a manner that the number of unsaturated bonds and its configuration affect the aggregate type (oil droplets, micelles, vesicles) and size [52, 53]. Studies supports the idea that lipid membrane surfaces can facilitate the structural transformation of a polypeptide chain into an unfolded conformation required to aggregate formation [54]. Thus, it is tempting to speculate that unsaturated fatty acids released under inflammatory conditions could interact with immature metal deficient SOD1 species enhancing the formation of aggregates. This effect could be mediated indirectly through the formation of micellar/vesicular states and/or directly through hydrophobically oriented interactions between the *cis*-unsaturated fatty acid and the protein.

Aggregation propensities of SOD1 have been correlated to thiol cross-linking reactions involving free Cys residues in the protein [41, 55]. Thus we sought to examine the participation of specific free thiol groups in DHA-induced apo-SOD1 oligomerization. As mentioned earlier, SDS-PAGE analysis showed that HMW aggregates are sensitive to thiol reductants, indicating that large SOD1 aggregates were disulfide-cross-linked. Analyses of the relative contribution of Cys-6 and Cys-111 evaluated by site-directed mutagenesis confirmed the requirement of these residues for the aggregation. Noticeably, WT and G93A SOD1 showed distinct Cys requirements for aggregation. For the WT, aggregation was abolished in both C6S and C111S mutants, while for the G93A aggregation was not observed only with the C6S mutant. Thus, Cys-6 seems to be a critical residue involved in DHA-induced aggregation of both WT and G93A SOD1.

Cys-111 is a solvent exposed residue known to be easily oxidized [45]. On the other hand, Cys-6 is found tightly packed in the interior of the beta-barrel in native homodimeric metalated forms of SOD1. It has been demonstrated that in the apo state, the protein becomes structurally highly disordered [41]. In this situation the protein experiences a broad range of conformations allowing Cys-6 to become more solvent accessible [41]. The aggregation observed for C111S mutant of G93A supports the hypothesis that DHA causes structural alterations increasing Cys-6 accessibility for intermolecular disulfide cross-linking. Interestingly, Antimone *et al*. [56] showed that Cys-6 is the primarily site of palmitoylation in immature SOD1. Thus while Cys-111 seems to be the preferential site to undergo oxidation [44, 46] and glutathionylation [57], Cys-6 seems to be a critical site for protein lipidation and also for DHA-induced HMW aggregate formation.

In summary, our study shows a distinct pattern of SOD1 oligomerization *in vitro* whereby DHA promotes SOD1 HMW aggregate species formation, whereas DHAOOH leads to aberrant covalent protein dimerization. It is noteworthy that both modes of oligomerization leading to HMW aggregate or dimeric species are found in cellular and rodent models of ALS [47]. Given the potential involvement of WT SOD1 in sporadic ALS [9], it is also relevant that DHA and DHAOOH lead to WT apo-SOD1 oligomerization to the same extent as the G93A mutant protein. Here we propose a mechanism whereby DHA interaction with apo-SOD1 favors protein hydrophobic patches exposure and Cys-6 accessibility to form intermolecular disulfide cross-links and consequent HMW species assembling. Aggregates formed in the presence of DHA have an amorphous morphology with similar characteristics of those found in ALS patients [49] and also recently found under oxidative conditions [36, 43]. Dimerization mechanism induced by DHAOOH was not further explored in this study. However, it is clear from our data that DHAOOH enhances the formation of non-native dimers strongly resistant to thiol reductants. Covalent SOD1 dimers involving di-tryptophan crosslinks has been already described [12]. We are currently investigating whether similar dimers are formed upon DHAOOH incubation. Since reactive oxygen species appears to contribute to ALS pathogenesis, oligomerization (aggregation and dimer formation) induced by DHA and its oxidized counterpart (the hydroperoxides) warrants further investigation to determine its relevance in the disease context.

## Supporting Information

S1 Fig DHAOOH induced apo-SOD1 dimer formation. SDS-PAGE under reducing condition of apo-SOD1 G93A (10 M) incubated in the absence and presence of DHAOOH. After 24 h incubation, guanidine (2 M) and DTT (166 mM) were added and incubated for 4 h. Thereafter, 200 mM of iodoacetamide was added and incubated overnight. The incubations were washed repeatedly and concentrated using a Amicon Ultra-Centrifugal Filter (30 kDa).
(TIF)

## References

[1] Bruijn L, Miller T, Cleveland D. Unraveling the mechanisms involved in motor neuron degeneration in ALS. Annu Rev Neurosci. 2004; 27: 723–749. 15217349 10.1146/annurev.neuro.27.070203.144244

[2] Ferraiuolo L, Kirby J, Grierson AJ, Sendtner M, Shaw PJ. Molecular pathways of motor neuron injury in amyotrophic lateral sclerosis. Nat Rev Neurol. 2011; 7: 616–630. 10.1038/nrneurol.2011.152 22051914

[3] Rosen DR, Siddique T, Patterson D, Figlewicz DA, Sapp P, Hentati A, et al Mutations in Cu/Zn superoxide dismutase gene are associated with familial amyotrophic lateral sclerosis. Nature. 1993; 362: 59–62. 8446170 10.1038/362059a0

[4] Cleveland DW, Rothstein JD. From charcot to lou gehrig: deciphering selective motor neuron death in als. Nat Rev Neurosci. 2001; 2: 806–819. 11715057 10.1038/35097565

[5] Valentine JS, Doucette PA, Zittin Potter S. Cooper-Zinc Superoxide Dismutase and Amyotrophic Lateral Sclerosis. Ann Rev Biochem. 2005; 74: 563–593. 15952898 10.1146/annurev.biochem.72.121801.161647

[6] Banci L, Bertini I, Boca M, Girotto S, Martinelli M, Valentine JS, et al SOD1 and Amyotrophic Lateral Sclerosis: Mutations and Oligomerization. PLoS One. 2008; 3: e1677 10.1371/journal.pone.0001677 18301754 PMC2250751

[7] Banci L, Bertini I, Durazo A, Girotto S, Gralla EB, Martinelli M, et al Metal-free superoxide dismutase forms soluble oligomers under physiological conditions: A possible general mechanism for familial ALS. Proc Natl Acad Sci U S A. 2007; 104: 11263–11267. 17592131 10.1073/pnas.0704307104 PMC1899188

[8] Lelie HL, Liba A, Bourassa MW, Chattopadhyay M, Chan PK, Gralla EB, et al Copper and Zinc Metallation Status of Copper-Zinc Superoxide Dismutase from Amyotrophic Lateral Sclerosis Transgenic Mice. J Biol Chem. 2011; 286: 2795–2806. 10.1074/jbc.M110.186999 21068388 PMC3024775

[9] Furukawa Y. Protein aggregates in pathological inclusions of amyotrophic lateral sclerosis. Amyotroph Lat Scl. 2012: 335–356.

[10] Rakhit R, Cunningham P, Furtos-Matei A, Dahan S, Qi X-F, Crow JP, et al Oxidation-induced Misfolding and Aggregation of Superoxide Dismutase and Its Implications for Amyotrophic Lateral Sclerosis. J Biol Chem. 2002; 277: 47551–47556. 12356748 10.1074/jbc.M207356200

[11] Taylor DM, Gibbs BF, Kabashi E, Minotti S, Durham HD, Agar JN. Tryptophan 32 potentiates aggregation and cytotoxicity of a copper/zinc superoxide dismutase mutant associated with familial amyotrophic lateral sclerosis. J Biol Chem. 2007; 282: 16329–16335. 17389599 10.1074/jbc.M610119200

[12] Medinas DB, Gozzo FC, Santos LFA, Iglesias AH, Augusto O. A ditryptophan cross-link is responsible for the covalent dimerization of human superoxide dismutase 1 during its bicarbonate-dependent peroxidase activity. Free Radic Biol Med. 2010; 49: 1046–1053. 10.1016/j.freeradbiomed.2010.06.018 20600836

[13] Perrin RJ, Woods WS, Clayton DF, George JM. Exposure to long chain polyunsaturated fatty acids triggers rapid multimerization of synucleins. J Biol Chem. 2001; 276: 41958–41962. 11553616 10.1074/jbc.M105022200

[14] Broersen K, van den Brink D, Fraser G, Goedert M, Davletov B. a-Synuclein Adopts an a-Helical Conformation in the Presence of Polyunsaturated Fatty Acids To Hinder Micelle Formation. Biochem. 2006; 45: 15610 17176082 10.1021/bi061743l

[15] Johansson AS, Garlind A, Berglind-Dehlin F, Karlsson G, Edwards K, Gellerfors P, et al Docosahexaenoic acid stabilizes soluble amyloid-beta protofibrils and sustains amyloid-beta-induced neurotoxicity in vitro. Febs J. 2007; 274: 990–1000. 17227385 10.1111/j.1742-4658.2007.05647.x

[16] Martins IC, Kuperstein I, Wilkinson H, Maes E, Vanbrabant M, Jonckheere W, et al Lipids revert inert A beta amyloid fibrils to neurotoxic protofibrils that affect learning in mice. EMBO J. 2008; 27: 224–233. 18059472 10.1038/sj.emboj.7601953 PMC2206134

[17] Aisenbrey C, Borowik T, Byström R, Bokvist M, Lindström F, Misiak H, et al How is protein aggregation in amyloidogenic diseases modulated by biological membranes? Eur Biophys J. 2008; 37: 247 18030461 10.1007/s00249-007-0237-0

[18] Tsiroulnikov K, Shchutskaya Y, Muronetz V, Chobert J-M, Haertlé T. Phospholipids influence the aggregation of recombinant ovine prions: From rapid extensive aggregation to amyloidogenic conversion. Biochim Biophys Acta. 2009; 1794: 506 10.1016/j.bbapap.2008.12.002 19124088

[19] Choi I, In Yang Y, Song HD, Lee JS, Kang T, Sung JJ, et al Lipid molecules induce the cytotoxic aggregation of Cu/Zn superoxide dismutase with structurally disordered regions. Biochim Biophys Acta 2011; 1812: 41–48. 10.1016/j.bbadis.2010.09.003 20837142

[20] De Franceschi G, Frare E, Pivato M, Relini A, Penco A, Greggio E, et al Structural and morphological characterization of aggregated species of α-synuclein induced by docosahexaenoic acid. J Biol Chem. 2011; 286: 22262–22274. 10.1074/jbc.M110.202937 21527634 PMC3121372

[21] Zhang M, Yang F Jr, Yang F, Chen J, Zheng C-Y, Liang Y. Cytotoxic aggregates of α-lactalbumin induced by unsaturated fatty acid induce apoptosis in tumor cells. Chem Biol Interact. 2009; 180: 131–142. 10.1016/j.cbi.2009.03.019 19497410

[22] Kim Y-J, Nakatomi R, Akagi T, Hashikawa T, Takahashi R. Unsaturated fatty acids induce cytotoxic aggregate formation of amyotrophic lateral sclerosis-linked superoxide dismutase 1 mutants. J Biol Chem. 2005; 280: 21515–21521. 15799963 10.1074/jbc.M502230200

[23] Kim H-Y. Novel Metabolism of Docosahexaenoic Acid in Neural Cells. J Biol Chem. 2007; 282: 18661–18665. 17488715 10.1074/jbc.R700015200

[24] Bazan NG, Molina MF, Gordon WC. Docosahexaenoic Acid Signalolipidomics in Nutrition: Significance in Aging, Neuroinflammation, Macular Degeneration, Alzheimer's, and Other Neurodegenerative Diseases. In: Cousins RJ, Bier DM, Bowman BA, editors. Annu Rev Nutr. Annual Review of Nutrition. 312011. p. 321–351. 10.1146/annurev.nutr.012809.104635 PMC3406932 21756134

[25] Orr SK, Palumbo S, Bosetti F, Mount HT, Kang JX, Greenwood CE, et al Unesterified docosahexaenoic acid is protective in neuroinflammation. J Neurochem. 2013; 127: 378–393. 10.1111/jnc.12392 23919613 PMC4068707

[26] Shibata N, Kakita A, Takahashi H, Ihara Y, Nobukuni K, Fujimura H, et al Increased expression and activation of cytosolic phospholipase A2 in the spinal cord of patients with sporadic amyotrophic lateral sclerosis. Acta Neuropathol. 2010; 119: 345–354. 10.1007/s00401-009-0636-7 20066429

[27] McGeer PL, McGeer EG. Inflammatory processes in amyotrophic lateral sclerosis. Muscle and Nerve. 2002; 26: 459–470. 12362410 10.1002/mus.10191

[28] Philips T, Robberecht W. Neuroinflammation in amyotrophic lateral sclerosis: Role of glial activation in motor neuron disease. Lancet Neurol. 2011; 10: 253–263. 10.1016/S1474-4422(11)70015-1 21349440

[29] Kiaei M, Kipiani K, Petri S, Choi D-K, Chen J, Calingasan NY, et al Integrative role of cPLA2 with COX-2 and the effect of non-steriodal anti-inflammatory drugs in a transgenic mouse model of amyotrophic lateral sclerosis. J Neurochem. 2005; 93: 403–411. 15816863 10.1111/j.1471-4159.2005.03024.x

[30] Derogis PBMC, Freitas FP, Marques ASF, Cunha D, Appolinário PP, de Paula F, et al The development of a specific and sensitive LC-MS-based method for the detection and quantification of hydroperoxy- and hydroxydocosahexaenoic acids as a tool for lipidomic analysis. PLoS One. 2013; 8: e77561 10.1371/journal.pone.0077561 24204871 PMC3812029

[31] Serhan CN, Petasis NA. Resolvins and protectins in inflammation resolution. Chem Rev. 2011; 111: 5922–5943. 10.1021/cr100396c 21766791 PMC3192290

[32] Miyamoto S, Martinez GR, Rettori D, Augusto O, Medeiros MHG, Di Mascio P. Linoleic acid hydroperoxide reacts with hypochlorous acid, generating peroxyl radical intermediates and singlet molecular oxygen. Proc Natl Acad Sci U S A. 2006; 103: 293–298. 16387855 10.1073/pnas.0508170103 PMC1326168

[33] Medinas DB, Toledo JJC, Cerchiaro G, do-Amaral AT, de-Rezende L, Malvezzi A, et al Peroxymonocarbonate and Carbonate Radical Displace the Hydroxyl-like Oxidant in the Sod1 Peroxidase Activity under Physiological Conditions. Chem Res Toxicol. 2009; 22: 639–648. 10.1021/tx800287m 19243126

[34] Benov LT, Beyer WF Jr, Stevens RD, Fridovich I. Purification and characterization of the Cu,Zn SOD from Escherichia coli. Free Radic Biol Med. 1996; 21: 117–121. 8791100 10.1016/0891-5849(95)02217-1

[35] Nilsson MR. Techniques to study amyloid fibril formation in vitro. Methods. 2004; 34: 151–160. 15283924 10.1016/j.ymeth.2004.03.012

[36] Coelho FR, Iqbal A, Linares E, Silva DF, Lima FS, Cuccovia IM, et al Oxidation of the Tryptophan 32 Residue of Human Superoxide Dismutase 1 Caused by Its Bicarbonate-dependent Peroxidase Activity Triggers the Non-amyloid Aggregation of the Enzyme. J Biol Chem. 2014; 289: 30690–30701. 10.1074/jbc.M114.586370 25237191 PMC4215247

[37] Jonsson PA, Graffmo KS, Andersen PM, Brännström T, Lindberg M, Oliveberg M, et al Disulphide-reduced superoxide dismutase-1 in CNS of transgenic amyotrophic lateral sclerosis models. Brain. 2006; 129: 451–464. 16330499 10.1093/brain/awh704

[38] Chattopadhyay M, Durazo A, Sohn SH, Strong CD, Gralla EB, Whitelegge JP, et al Initiation and elongation in fibrillation of ALS-linked superoxide dismutase. Proc Natl Acad Sci U S A. 2008; 105: 18663–18668. 10.1073/pnas.0807058105 19022905 PMC2585484

[39] Furukawa Y, Kaneko K, Yamanaka K, O'Halloran TV, Nukina N. Complete loss of post-translational modifications triggers fibrillar aggregation of SOD1 in the familial form of amyotrophic lateral sclerosis. J Biol Chem. 2008; 283: 24167–24176. 10.1074/jbc.M802083200 18552350 PMC3259764

[40] Ding F, Dokholyan NV. Dynamical roles of metal ions and the disulfide bond in Cu, Zn superoxide dismutase folding and aggregation. Proc Natl Acad Sci U S A. 2008; 105: 19696–19701. 10.1073/pnas.0803266105 19052230 PMC2604981

[41] Banci L, Bertini I, Boca M, Calderone V, Cantini F, Girotto S, et al Structural and dynamic aspects related to oligomerization of apo SOD1 and its mutants. Proc Natl Acad Sci U S A. 2009; 106: 6980–6985. 10.1073/pnas.0809845106 19369197 PMC2678485

[42] Fu X, Zhang X, Chang Z. 4,4'-Dianilino-1,1'-binaphthyl-5,5'-sulfonate, a novel molecule having chaperone-like activity. Biochem Biophys Res Commun. 2005; 329: 1087–1093. 15752765 10.1016/j.bbrc.2005.01.164

[43] Mulligan VK, Kerman A, Laister RC, Sharda PR, Arslan PE, Chakrabartty A. Early Steps in Oxidation-Induced SOD1 Misfolding: Implications for Non-Amyloid Protein Aggregation in Familial ALS. J Biol Chem. 2012; 421: 631–652. 10.1016/j.jmb.2012.04.016 22542526

[44] Chen X, Shang H, Qiu X, Fujiwara N, Cui L, Li X-M, et al Oxidative Modification of Cysteine 111 Promotes Disulfide Bond-Independent Aggregation of SOD1. Neurochem Res. 2012; 37: 835–845. 10.1007/s11064-011-0679-8 22219129

[45] Cozzolino M, Amori I, Pesaresi MG, Ferri A, Nencini M, Carrì MT. Cysteine 111 Affects Aggregation and Cytotoxicity of Mutant Cu,Zn-superoxide Dismutase Associated with Familial Amyotrophic Lateral Sclerosis. J Biol Chem. 2008; 283: 866–874. 18006498 10.1074/jbc.M705657200 PMC2842925

[46] Fujiwara N, Nakano M, Kato S, Yoshihara D, Ookawara T, Eguchi H, et al Oxidative Modification to Cysteine Sulfonic Acid of Cys111 in Human Copper-Zinc Superoxide Dismutase. J Biol Chem. 2007; 282: 35933–35944. 17913710 10.1074/jbc.M702941200

[47] Shaw BF, Valentine JS. How do ALS-associated mutations in superoxide dismutase 1 promote aggregation of the protein? Trends Biochem Sci. 2007; 32: 78–85. 17208444 10.1016/j.tibs.2006.12.005

[48] Turell L, Carballal S, Botti H, Radi R, Alvarez B. Oxidation of the albumin thiol to sulfenic acid and its implications in the intravascular compartment. Braz J Med Biol Res. 2009; 42: 305–311. 19330257 10.1590/s0100-879x2009000400001

[49] Kerman A, Liu HN, Croul S, Bilbao J, Rogaeva E, Zinman L, et al Amyotrophic lateral sclerosis is a non-amyloid disease in which extensive misfolding of SOD1 is unique to the familial form. Acta Neuropathol. 2010; 119: 335–344. 10.1007/s00401-010-0646-5 20111867

[50] Kato S, Takikawa M, Nakashima K, Hirano A, Cleveland DW, Kusaka H, et al New consensus research on neuropathological aspects of familial amyotrophic lateral sclerosis with superoxide dismutase 1 (SOD1) gene mutations: Inclusions containing SOD1 in neurons and astrocytes. Amyotroph Lat Scl. 2000; 1: 163–184. 10.1080/14660820050515160 11464950

[51] Hwang YM, Stathopulos PB, Dimmick K, Yang H, Badiei HR, Tong MS, et al Nonamyloid aggregates arising from mature copper/zinc superoxide dismutases resemble those observed in amyotrophic lateral sclerosis. J Biol Chem. 2010; 285: 41701–41711. 10.1074/jbc.M110.113696 20974846 PMC3009897

[52] Morigaki K, Walde P. Fatty acid vesicles. Current Opin Colloid Interface Sci. 2007; 12: 75–80.

[53] Namani T, Ishikawa T, Morigaki K, Walde P. Vesicles from docosahexaenoic acid. Colloids Surf B Biointerfaces. 2007; 54: 118–123. 16829059 10.1016/j.colsurfb.2006.05.022

[54] Gorbenko GP, Kinnunen PKJ. The role of lipid–protein interactions in amyloid-type protein fibril formation. Chem Phys Lipids. 2006; 141: 72–82. 16569401 10.1016/j.chemphyslip.2006.02.006

[55] Furukawa Y, Fu R, Deng H-X, Siddique T, O'Halloran TV. Disulfide cross-linked protein represents a significant fraction of ALS-associated Cu, Zn-superoxide dismutase aggregates in spinal cords of model mice. Proc Natl Acad Sci U S A. 2006; 103: 7148–7153. 16636274 10.1073/pnas.0602048103 PMC1447524

[56] Antinone SE, Ghadge GD, Lam TT, Wang L, Roos RP, Green WN. Palmitoylation of Superoxide Dismutase 1 (SOD1) Is Increased for Familial Amyotrophic Lateral Sclerosis-linked SOD1 Mutants. J Biol Chem. 2013; 288: 21606–21617. 10.1074/jbc.M113.487231 23760509 PMC3724620

[57] Wilcox KC, Zhou L, Jordon JK, Huang Y, Yu Y, Redler RL, et al Modifications of Superoxide Dismutase (SOD1) in Human Erythrocytes: a possible role in amyotrophic lateral sclerosis. J Biol Chem. 2009; 284: 13940–13947. 10.1074/jbc.M809687200 19299510 PMC2679493

