# Supplementary figures and images for: Oligomerization of Cu,Zn-Superoxide Dismutase (SOD1) by Docosahexaenoic Acid and Its Hydroperoxides In Vitro: Aggregation Dependence on Fatty Acid Unsaturation and Thiols
Source: PLoS One. 2015 Apr 30;10(4):e0125146. doi: 10.1371/journal.pone.0125146 (PMC4415921; doi:10.1371/journal.pone.0125146)

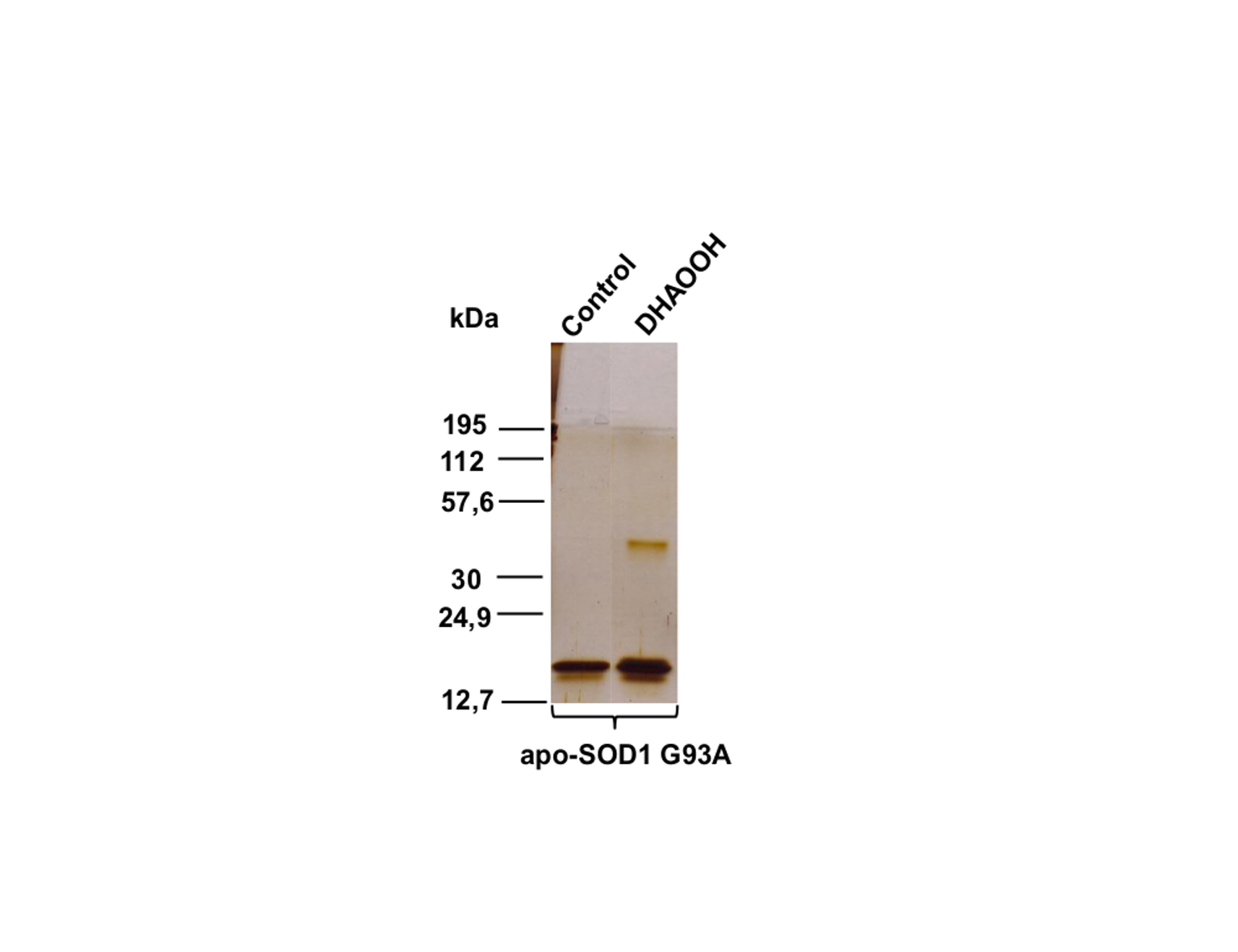

Supplement: S1 Fig — SDS-PAGE under reducing condition of apo-SOD1 G93A (10 M) incubated in the absence and presence of DHAOOH. After 24 h incubation, guanidine (2 M) and DTT (166 mM) were added and incubated for 4 h. Thereafter, 200 mM of iodoacetamide was added and incubated overnight. The incubations were washed repeatedly and concentrated using a Amicon Ultra-Centrifugal Filter (30 kDa). (TIF) [file pone.0125146.s001.tif]
